# High-viscosity bone cement for vertebral compression fractures: a prospective study on intravertebral diffusion and leakage of bone cement

**DOI:** 10.1186/s12891-020-03613-7

**Published:** 2020-09-02

**Authors:** Meiyong Wang, Qunhua Jin

**Affiliations:** 1 Qilu Hospital, Cheeloo College of Medicine, Shandong University, Jinan, 250012 Shandong China; 2 The third staff Hospital of Baogang group, 014010, Baotou, Inner Mongolia Autonomous Region China; 3 General hospital of Ningxia Medical University, Yinchuan, China

**Keywords:** Percutaneous vertebroplasty, Bone cement, leakage, osteoporosis, elderly, pain, diffusion

## Abstract

**Background:**

Bone cement leakage causes severe complication following percutaneous vertebroplasty. This study probed the diffusion and leakage status of bone cement injected within diverged time duration, so as to find the optimal injection time for bone cement.

**Methods:**

A total of 70 patients with osteoporotic vertebral compression fractures with a symptom of low back pain, who underwent treatment at hospital were enrolled in this study. Patients were randomized into three groups: < 180 s, 180–300, and > 300 s of injection time duration from the beginning to the completion of the injection. The scenarios of vertebral bone cement leakage and diffusion were inspected using postoperative CT.

**Results:**

The diffusion coefficient was higher in group A than in group B whereas it was higher in group B than in group C, but without statistical significance among the three groups. The leakage rate was without statistical significance among the three groups. The injection time of bone cement was negatively correlated with the diffusion coefficient, at the correlation coefficient of − 0.253.

**Conclusions:**

The diffusion coefficient of high-viscosity bone cement is negatively correlated with the injection time, and the leakage rate of high-viscosity bone cement does not reduce with the prolongation of injection time.

## Background

Osteoporosis is one of the most common diseases of the elderly, and it results in considerable pain and decreased quality of life if not treated appropriately [1].Leakage of bone cement into the spinal canal or cardiovascular system could generate severe complications, such as nerve compression or embolism, and even cause death [2–4].

High-viscosity bone cement was applied to reduce the leakage risk [4–7], with the advantage of optionally long injection time, allowing surgeons to determine whether the injection would be continued before cement leakage takes place, thus prominently improving operative safety [8].

Injection timing of bone cement is the direct impact factor for leakage. Baroud et al. [5] proposed cement leakage ceased completely when cement was delivered after 10 min, with good viscosity, and not prone to leakage [9]. Surgeons prefer injecting cement earlier, which causes rapid diffusion and leakage, to avoid procedure failure; however, delayed injection could raise difficulty due to the setting of cement [10]. Therefore, a precise timing of bone cement injection is critical, which should avoid leakage while cement spread more uniformly. The cement fills more uniformly is an essential element in the reconstruction of stiffness and biomechanical balance of the fractured vertebral body [11, 12].

At present, most studies of bone cement focus on the therapeutic effect while rarely on the injection time after starting the mixing or on diffusion within the vertebral body.

### Objective

Bone cement leakage causes severe complication following percutaneous vertebroplasty. This study probed the diffusion and leakage status of bone cement injected within diverged time duration, so as to find the optimal injection time of bone cement.

## Methods

### Study design: this was a prospective case analysis

#### Study time and institution

A total of 70 cases of thoracolumbar vertebral compression fractures with a symptom of low back pain, who underwent treatment between August 2014 and April 2016 at Xiyuan Hospital, Chinese Academy of Traditional Chinese Medicine,and was approved by the hospital’s ethics Committee.Data were enrolled in this study (Table 1). These patients underwent PVP, and the bone mineral density (BMD) was measured routinely before operation, in addition to x-ray imaging of the anteroposterior and lateral positions of the thoracolumbar spine and magnetic resonance imaging (MRI). The inclusion criteria were as follows: (1) patients aged more than 60 years, with osteopenia displayed by BMD measurement; (2) patients with fresh fractures confirmed by MRI, which was shown by the x-ray examination as a single vertebral compression fracture, with the degree of vertebral compression no more than 75%; (3) patients whose fracture occurred spontaneously or was caused by minor trauma; (4) patients without any infection within the 15 cm range of the puncture position; (5) patients without cardiac pulmonary, hepatic, or renal failure; and (6) patients without coagulopathy or bleeding tendency. All patients were informed of the treatment modality after admission to hospital, and before surgery, they were informed that their follow-up information would be included in this study. Excluded cases comprised patients with concurrent spinal cord compression or nerve injury; or spinal stenosis exceeding 30%; or concomitant spinal tuberculosis, tumor, and rheumatoid arthritis.

**Table 1:** Patients Characteristics or Patients base line data

*Item*	*Group A (< 180 s)*	*Group B (180–300 s)*	*Group C (> 300 s)*
*Case number*	*24*	*23*	*23*
*Male/Female*	*9/15*	*7/16*	*6/17*
*Age (year)*	*76.6 ± 7.9*	*74.7 ± 6.8*	*74.2 ± 8.1*
*Mean time duration between disease onset and surgery (day)*	*4*	*4*	*5*
*Diseased vertebral bodies*			
*T7*	*1*		*1*
*T8*			
*T9*	*1*	*1*	*1*
*T10*		*2*	*2*
*T11*	*4*	*7*	*4*
*T12*	*6*	*9*	*3*
*L1*	*7*	*2*	*5*
*L2*	*1*	*2*	*2*
*L3*	*3*	*2*	*4*
*L4*	*1*		*1*

#### Study groups

The 70 eligible patients, with totally diseased vertebral bodies, visiting the hospital during this period were randomly allocated into three groups by the computer software based on their hospitalization numbers: group A (injection time duration < 180 s); group B (injection time duration at the range of 180–300 s); groups C (injection time duration > 300 s). The specific baseline information for each group was as Table 1.

#### Materials

Acrylic resin bone cement was purchased from TEKNIMED S.A., France(BP 6065502 VIC EN BIGORRE Cedex); bone cement mixer and vertebroplasty instrument (expansion type) were purchased from ZhongShan ShiYiTang Medical Equipment Co., Ltd. [Specification and model: 15 mL, PKP-I; product standard number: YZB/Guangdong 0574–2010; product registration certificate number: Guangdong Food and Drug Administration (quasi) 2011 NO. 2100306 (more)]; and computed tomography (CT) system was purchased from General Electric Company (Model: Lightspeed VCT).

### Surgical technique

#### Vertebroplasty

The diseased vertebral body was located using C arm fluoroscopy, and the point and angle of needle insertion were determined at the lateral position. The needle was inserted from the lateral superior direction to the vertebral pedicle (the left side was at 10 o’clock direction to the vertebral pedicle while the right side was at 2 o’clock direction). An incision was made through which the puncture needle was inserted, followed by the working cannula. High-viscosity bone cement was mixed and prepared by the surgical first assistant, whereas the second assistant started to count time and write records.The polymethyl methacrylate powder is put into a container. Liquid methyl methacrylate monomer was added to the powder and the mixture was mixed to a toothpaste-like consistency.The operations are carried out according to the instructions. After the cement was prepared, it was loaded into the rotary pressure pump, followed by slow injection into the vertebral body by the surgeon using C-arm fluoroscopy, a turn of the pump handle was applied appromimately 0.3 cc. The injection was immediately stopped and the time was counted when bone cement reached the posterior wall of the vertebral body displayed under fluoroscopy. The injection volume of bone cement was visible via the scale on the filling machine and recorded. After the complete setting of cement, the working cannula was removed, and the wound was pressed for hemostasis and bound up.

#### Main outcome measures

The patients’ vital signs and complications of cement leakage were intensively monitored after surgery. CT inspection was performed for all patients 3 days after surgery. Three dimensional (3D) images of the vertebral body and bone cement were reconstructed on an image workstation by another group of medical staff via 3D imaging and volume rendering of CT data, and the measurement of bone cement and vertebral body was enabled via relevant functions of the workstation.

### Measurement of the diffused cement volume within the vertebral body

The diffusion volume of bone cement is a three-dimensional structure formed by wrapping bone trabeculae and their gaps with bone cement after bone cement is injected into vertebral body. The diffusion capacity of bone cement injected into the vertebral body was reflected by diffusion coefficient: diffusion coefficient = diffusion volume/injection volume of cement. The postoperative image of the vertebral body was retrieved from the workstation. Subsequently, the boundary of cement diffusion was outlined and other tissues were deleted except for bone cement, prior to the acquisition of the diffusion volume of bone cement after several fractionations.

Analysis of bone cement leakage: Postoperative 3D images of the vertebral body and bone cement rendered on the image workstation were checked to record and analyze the leakage situation of bone cement, including paravertebral, intraspinal, and intervertebral space leakages. Subsequently, the leakage rate of bone cement was calculated for each group.

#### Statistical analysis

The statistical analysis was conducted using statistical software SPSS16.0 (SPSS Inc., USA). Enumeration and measurement data were expressed, respectively, with frequency/percentage (n/%) and mean ± standard deviation (x ± s). Specific analysis included the following: (1) the mean and standard deviation of the injection and diffusion volumes were calculated for the three groups, followed by the normal distribution test; (2) the diffusion coefficient of bone cement among the three groups was compared by one-way analysis of variance; (3) the leakage rates among the three groups were compared using the chi-square test; (4) Pearson correlation analysis was used to measure the correlation of bone cement injection time with diffusion coefficient for the three individual groups; and (5) the analysis power was preset (α = 0.05), and a *P* value < 0.05 was designated as statistically significant.

## Results

### Diffusion coefficient of bone cement in different time periods

Bone cement injection volume, diffusion volume, and diffusion coefficient of the three groups are listed in Table 2. The diffusion coefficient was higher in group A than in group B while higher in group B than in group C, but without statistical significance among the three groups (*P* = 0.113).

**Table 2:** Bone cement distribution in the three groups

*Item*	*Group A (< 180 s)*	*Group B (180–300 s)*	*Group C (> 300 s)*	*P value*
*Injection volume (x ± s, mL)*	*3.80 ± 0.43*	*3.93 ± 0.36*	*4.02 ± 0.33*	
*Diffusion volume (x ± s, mL)*	*7.90 ± 0.52*	*7.74 ± 0.49*	*7.69 ± 0.57*	
*Diffusion coefficient*	*2.11 ± 0.32*	*1.99 ± 0.25*	*1.92 ± 0.21*	*0.113*
*Leakage case number*	*8*	*5*	*4*	
*Leakage rate (n/%)*	*33.3%*	*21.7%*	*17.4%*	*0.418*
*Complications of leakage*	*None*	*None*	*None*	
*Injection time (x ± s, s)*	*144.6 ± 23.1*	*241.5 ± 29.5*	*344.3 ± 32.1*	
*correlation coefficient*	-0.253			*0.04*

Leakage rate of bone cement at different time periods.

The leakage rate of bone cement was 33.3% in group A, 21.7% in group B, and 17.4% in group C, with no significant difference between group A and group C (*P* = 0.418).

### Correlation of bone cement injection time with diffusion coefficient

Bone cement injection time was negatively correlated with diffusion coefficient within a certain time range, with the correlation coefficient of − 0.253 (*P* < 0.05).

## Discussion

The present study demonstrated that the diffusion capability and leakage rate of high-viscosity bone cement did not decrease with the prolonged injection time, whereas the diffusion capability was negatively correlated with the injection time. The mean diffusion coefficient of bone cement was higher in group A than in groups B and C, but without statistical significance (*P* = 0.113), indicating that the diffusion capability of high-viscosity bone cement in the vertebral body does not alleviate with prolonged time within a given time range, which could be because of the sticky phase of bone cement and stable property at this range.

The diffusion coefficient of bone cement was negatively correlated with injection time within a certain time range. The diffusion ranges varied with different injection time durations. The viscosity of bone cement is presumed to elevate with time prolongation following preparation, and early injected cement, diffusing to a certain range, inhibits the diffusion of succeeding cement, giving rise to a reduced diffusion range.

In the present study, the leakage status of high-viscosity bone cement was compared among the three groups with different injection times, and the findings revealed that although prolonged injection could reduce leakage, the difference was not statistically significant. Therefore, it is irrational to purposely prolong bone cement injection to reduce the leakage rate of bone cement in clinical practice. High-viscosity bone cement is injectable in 1–2 min after starting mixing, with short waiting time and long operable time; therefore, the surgeon needs no estimation or prediction for injection time, greatly declining the leakage rate. However, prolonged work with bone cement offers enough time to a surgeon to decide how to inject bone cement into the vertebral body. Boger A. claimed that the injection of bone cement should be delayed as much as possible to prevent leakage and overflow [12]. The incidence rate of bone cement leakage has varied between 5 and 87% [13, 14], most of which was asymptomatic leakage and pulmonary cement embolism or spinal injury was rarely reported [15, 16]. Yeom [17]. and colleagues proposed CT imaging as the gold standard for diagnosing bone cement leakage, with a higher diagnostic value compared with x-ray imaging. However, in the present study, CT inspection was adopted for diagnosing leakage, at the rate of 24.3% (17/70), which was higher than that reported in previous studies.

Patients with single thoracolumbar vertebral fracture having similar clinical characteristics were enrolled in this study to ensure homogeneity of baseline features across the groups. Controllable variables were unified. For example, the same operating system and the same batch of bone cement were applied throughout the study, and surgery was performed by the same surgeon at the same room temperature, while bone cement was injected using a rotary pressure pump and mixed by the same surgical assistant, minimizing most interference factors.

This study had some limitations. The time range chosen for the study was limited, unable to cover the entire work time with bone cement. Moreover, the symptomatic recovery of patients after surgery was not assessed, thus results from this study are not complete. Outcome assessorsis always the same person.

## Conclusions

In brief, the diffusion capability of high-viscosity bone cement is likely not reduced with the prolongation of injection time within a certain time range, showing the negative correlation, and the leakage rate of high-viscosity bone cement is likely not reduced with the prolongation of injection time. The injection time of high viscosity bone cement should be as early as possible.

## Supplementary information

**Additional file 1.**

## Data Availability

All the authors agree to use. The original data has been provided. All data generated or analysed during this study are included in this published article [and its supplementary information files].

## Abbreviations

PVP: Percutaneous vertebroplasty
BMD: Bone mineral density
MRI: Magnetic resonance imaging
3D: Three dimensional
