# Supplementary material for: High-viscosity bone cement for vertebral compression fractures: a prospective study on intravertebral diffusion and leakage of bone cement
Source: BMC Musculoskelet Disord. 2020 Sep 2;21:589. doi: 10.1186/s12891-020-03613-7 (PMC7469425; doi:10.1186/s12891-020-03613-7)
Supplement: Supplementary file 1 — Additional file 1. [file 12891_2020_3613_MOESM1_ESM.doc]

| *Gender* | *Age* | *Number* | | | | *Diseased vertebral bodies* | *Diffusion coefficient* | *njection volume* | | *Diffusion volume* | *njection time* | |  | |
| --- | --- | --- | --- | --- | --- | --- | --- | --- | --- | --- | --- | --- | --- | --- |
|  | |
|  | |
| male | 72 | 154411 | | | | L2 | 1.74 | 4.7 | | 8.2 | 304 | |  | |
| female | 82 | 155332 | | | | L1 | 2.15 | 4.1 | | 8.8 | 399 | |  | |
| male | 69 | 140375 | | | | L2 | 1.98 | 4 | | 7.9 | 348 | |  | |
| male | 87 | 157642 | | | | L3 | 1.74 | 4.2 | | 7.3 | 381 | | 1 | |
| female | 80 | 0106608 | | | | T12 | 2 | 4.2 | | 8.4 | 354 | |  | |
| female | 77 | 0159009 | | | | L1 | 1.84 | 3.7 | | 6.8 | 420 | |  | |
| female | 86 | 0161128 | | | | T10 | 1.94 | 3.6 | | 7 | 307 | |  | |
| male | 70 | 0161289 | | | | L3 | 2 | 3.7 | | 7.4 | 324 | | 1 | |
| female | 62 | 0161322 | | | | L1 | 1.82 | 3.9 | | 7.1 | 321 | |  | |
| female | 73 | 0147706 | | | | T11 | 2.29 | 3.7 | | 8.5 | 335 | | 1 | |
| female | 58 | 0162120 | | | | T11 | 1.61 | 4.4 | | 7.1 | 387 | |  | |
| female | 61 | 0139574 | | | | T8 | 1.92 | 3.8 | | 7.3 | 356 | |  | |
| female | 63 | 0162864 | | | | L1 | 1.67 | 4.5 | | 7.5 | 370 | |  | |
| male | 80 | 0163080 | | | | T10 | 2.28 | 3.6 | | 8.2 | 342 | |  | |
| female | 78 | 0123757 | | | | L3 | 1.5 | 4.6 | | 6.9 | 310 | |  | |
| female | 70 | 0163881 | | | | L3 | 1.8 | 4.1 | | 7.4 | 355 | |  | |
| female | 75 | 0164421 | | | | T12 | 1.8 | 4.4 | | 7.9 | 346 | |  | |
| female | 69 | 0164591 | | | | T11 | 2.21 | 3.8 | | 8.4 | 311 | |  | |
| female | 78 | 0165296 | | | | L1 | 2 | 4 | | 8 | 305 | |  | |
| female | 82 | 0161605 | | | | T12 | 1.79 | 3.9 | | 7 | 321 | | 1 | |
| female | 79 | 0128399 | | | | T7 | 2.23 | 3.5 | | 7.8 | 375 | |  | |
| female | 69 | 0167537 | | | | T11 | 2.03 | 3.9 | | 7.9 | 341 | |  | |
| male | 86 | 0169032 | | | | L4 | 1.88 | 4.3 | | 8.1 | 306 | |  | |
|  | 8.14 |  | | | |  | 0.2083312 | 0.332602 | | 0.567154 | 7918 | |  | |
| female | 69 | | 152008 | | | T12 | 7.8 | | 4.5 | 1.73 | 214 | |  | |
| male | 76 | | 154353 | | | L1 | 7.9 | | 3.7 | 2.14 | 199 | |  | |
| male | 80 | | 155491 | | | L1 | 8.2 | | 3.6 | 2.28 | 243 | |  | |
| female | 77 | | 155883 | | | L3 | 7.4 | | 3.4 | 2.18 | 291 | |  | |
| male | 68 | | 156401 | | | L1 | 6.9 | | 3.9 | 1.77 | 265 | |  | |
| female | 74 | | 156693 | | | L1 | 7.1 | | 3.9 | 1.82 | 194 | |  | |
| female | 65 | | 0158714 | | | L2 | 6.8 | | 4.4 | 1.55 | 238 | |  | |
| female | 77 | | 0158908 | | | T12 | 7.4 | | 4.1 | 1.8 | 244 | | 1 | |
| female | 74 | | 0160132 | | | L1 | 7.6 | | 3.8 | 2 | 207 | |  | |
| male | 60 | | 0161116 | | | L1 | 7 | | 4.4 | 1.59 | 277 | |  | |
| female | 72 | | 0161323 | | | L2 | 8.1 | | 3.9 | 2.08 | 263 | |  | |
| female | 75 | | 0094586 | | | L1 | 7.7 | | 4.6 | 1.67 | 281 | |  | |
| male | 78 | | 0161723 | | | L1 | 8.3 | | 3.9 | 2.13 | 236 | |  | |
| female | 83 | | 0162994 | | | T11 | 8.1 | | 3.3 | 2.45 | 218 | | 1 | |
| female | 72 | | 0163832 | | | T11 | 8 | | 4 | 2 | 272 | | 1 | |
| female | 82 | | 0165201 | | | T12 | 7.9 | | 4.1 | 1.93 | 238 | |  | |
| female | 79 | | 0079586 | | | L3 | 8.6 | | 4.3 | 2 | 197 | |  | |
| female | 88 | | 0166572 | | | T12 | 8.2 | | 3.5 | 2.34 | 221 | |  | |
| female | 61 | | 0166719 | | | T12 | 7.3 | | 4.2 | 1.74 | 273 | | 1 | |
| male | 77 | | 0166886 | | | T11 | 7.9 | | 4.1 | 1.93 | 235 | |  | |
| male | 71 | | 0167886 | | | L1 | 7.3 | | 3.6 | 2.03 | 287 | |  | |
| female | 82 | | 0106608 | | | T9 | 8.4 | | 3.7 | 2.27 | 217 | |  | |
| female | 79 | | 0169415 | | | T11 | 8.1 | | 3.4 | 2.38 | 244 | | 1 | |
|  | 6.8 | |  | | |  | 0.5 | |  |  | 5554 | |  | |
| female | 69 | | | 154872 | L1 | | 7.9 | | 3.9 | 2.03 | 106 |  | |  |
| male | 77 | | | 155012 | T12 | | 8 | | 3.2 | 2.5 | 141 |  | |  |
| male | 78 | | | 155242 | L3 | | 7.2 | | 3.6 | 2 | 104 |  | |  |
| male | 64 | | | 156021 | L1 | | 7.1 | | 4.1 | 1.73 | 136 |  | |  |
| female | 82 | | | 49256 | T12 | | 7.9 | | 3.6 | 2.19 | 172 | 1 | |  |
| female | 84 | | | 154690 | L4 | | 7.8 | | 3.3 | 2.36 | 166 |  | |  |
| female | 75 | | | 157224 | L1 | | 8.3 | | 3.9 | 2.13 | 125 | 1 | |  |
| male | 88 | | | 157642 | T12 | | 8.6 | | 3.4 | 2.53 | 166 |  | |  |
| male | 84 | | | 2E+06 | T11 | | 8.1 | | 3.7 | 2.19 | 143 |  | |  |
| male | 83 | | | 0146167 | L3 | | 8.3 | | 3.4 | 2.44 | 115 | 1 | |  |
| female | 73 | | | 0159445 | L2 | | 7.9 | | 4.3 | 1.84 | 177 | 1 | |  |
| female | 82 | | | 0096382 | T11 | | 8.4 | | 2.9 | 2.9 | 167 |  | |  |
| female | 80 | | | 0138925 | T11 | | 7.2 | | 3.8 | 1.89 | 161 |  | |  |
| female | 70 | | | 0161567 | T9 | | 6.8 | | 4.5 | 1.51 | 139 |  | |  |
| female | 80 | | | 0161871 | T7 | | 7.4 | | 3.6 | 2.06 | 121 |  | |  |
| male | 62 | | | 0163376 | T12 | | 7.9 | | 4.4 | 1.8 | 124 | 1 | |  |
| female | 63 | | | 0165745 | L1 | | 7.7 | | 4.5 | 1.71 | 154 |  | |  |
| female | 78 | | | 0128399 | T12 | | 8.5 | | 4.1 | 2.07 | 172 |  | |  |
| male | 62 | | | 0166220 | T11 | | 7.1 | | 3.9 | 1.82 | 148 |  | |  |
| female | 77 | | | 0119084 | T12 | | 8.5 | | 4.3 | 1.98 | 130 |  | |  |
| female | 80 | | | 0167009 | L1 | | 8.7 | | 3.5 | 2.49 | 175 | 1 | |  |
| female | 73 | | | 0166730 | L1 | | 7.9 | | 4.4 | 1.8 | 107 |  | |  |
| male | 88 | | | 0085222 | L1 | | 8.2 | | 3.4 | 2.41 | 164 | 1 | |  |
| female | 86 | | | 0168935 | L3 | | 8.3 | | 3.7 | 2.24 | 144 | 1 | |  |
|  | 7.895 | | |  |  | |  | |  | 0.322101 | 3106 |  | |  |
